# Help-seeking behaviour in primary care of men and women with a history of abuse: A Dutch cohort study

**DOI:** 10.1080/13814788.2022.2054985

**Published:** 2022-04-05

**Authors:** Anieck M. Lomans, Annemarie A. Uijen, Reinier P. Akkermans, Toine A. L. M. Lagro-Janssen, Doreth A. M. Teunissen

**Affiliations:** Department of Primary and Community Care, Gender and Women’s Health, Radboud University Medical Centre, Nijmegen, The Netherlands

**Keywords:** General practice/family medicine, general, trauma, psychological problems, social problems

## Abstract

**Background:**

Previous studies show an association between a history of abuse and higher care demand. However, studies in general practice regarding help-seeking behaviour by patients (mainly male patients) with a history of abuse are scarce.

**Objectives:**

To analyse help-seeking behaviour in general practice of men and women with a history of abuse.

**Methods:**

A cohort study using data from a Dutch primary care registration network from 2015 to 2019. We included all patients aged ≥ 18 years who indicated on a questionnaire that they did or did not have a history of abuse. We analysed differences in contact frequency, types of contact, reason for encounter and diagnoses between men and women with or without a history of abuse.

**Results:**

The questionnaire had a response rate of 59% and resulted in 11,140 patients, of which 1271 indicated a history of abuse. Men and women with a history of abuse contact the general practitioner (GP) 1.5 times (95% CI 1.42–1.60) more often than men and women without a history of abuse, especially for psychological (rate ratio 1.97, 95% CI 1.79–2.17) and social (rate ratio 1.93, 95% CI 1.68–2.22) problems. Moreover, when diagnosed with a psychological or social problem, patients with a history of abuse contact the GP twice more often for these problems.

**Conclusion:**

Compared to men and women without a history of abuse, men and women with a history of abuse visit their GP more often, particularly for psychological and social problems.

Key messages

- Abuse affects help-seeking behaviour of both men and women.
- Men and women with a history of abuse contact their GP 1.5 times more often.
- A history of abuse increases GP contacts for psychological and social problems for both men and women.

## Introduction

Research has shown that abuse can lead to adverse long-term effects on mental and physical health for both men and women [1–9]. Abuse, which in this article is defined as psychological, physical and/or sexual abuse, increases the risk for disease conditions and risky behaviours, such as depression, panic disorder, posttraumatic stress syndrome, coronary heart disease, diabetes, obesity, alcoholism and smoking [1–4]. This leads to a higher contact frequency with medical services and higher health care costs in patients who experienced abuse in the past [3,5–7,10,11].

Recognition of health problems related to a history of abuse can be complex [12]. Since the experience covers a sensitive topic, victims may feel shame or are afraid it will impact their identity and may therefore not bring it up themselves [2]. Physicians can fear that discussing abuse is too personal even for the doctor–patient relationship [2,13]. Furthermore, victims may not relate certain health problems to abuse.

The general practitioner (GP) can play an important role in recognising and managing a history of abuse. Unfortunately, research showed that only one out of ten female victims of intimate partner violence (IPV) is known by their GP [14]. Recognition rates for men are unknown. Studies on help-seeking behaviour of female victims in general practice show that they visit their GP almost twice as often compared to non-victims, often concerning social problems, substance abuse and reproductive health problems [5–7].

These results indicate that a history of abuse in women leads to a higher care demand. For men, less research has been done on the relation between a history of abuse and help-seeking behaviour. Knowing that a history of abuse is still poorly recognised, we aim to investigate the difference in help-seeking behaviour and the reason to contact the general practice between patients with or without a history of abuse, explicitly including men as well. We will analyse differences in contact frequency, type of contact, reason for encounter and diagnoses between patients with or without a history of abuse and whether the effect of abuse on health care is related to gender. Increasing the knowledge in help-seeking behaviour and reasons for encountering general practice may help in earlier recognition and lowering disease burden in patients with a past of physical, psychological or sexual abuse.

## Methods

### Data source

We performed a cohort study using data from the Family Medicine Network (FaMe-Net), a primary health care registration network in the Netherlands [15]. Within this network all encounters of patients with general practice are registered since 1971. In the last 20 years, five practices participated with 31,983 patients. GPs routinely code each episode of care. An episode of care is defined as a health problem in an individual from the first encounter until the completion of the last encounter. For all encounters, the GP registers the patient’s reason for encounter (RFE), GP’s diagnosis and GP’s interventions according to the International Classification of Primary Care (ICPC) [15,16].

An episode of care and its associated ICPC codes represent an individual’s help-seeking behaviour. The first RFE of an episode is the ‘literal uttered reason’ of the patient why he/she contacted the GP, translated into an ICPC code by the GP. RFE’s can be symptoms, complaints or a request for an intervention. It reflects the patient’s personal needs, expectations and priorities around their health problem. Each episode of care has one final diagnosis made by the GP (this definitive diagnosis can change over time; for example the diagnosis can change from ‘tiredness’ to ‘anaemia’ when the results of laboratory testing are known). Within an episode of care, there may be several contacts. The GPs have clear agreements for coding RFE, diagnoses and interventions, as the participating GPs have regular meetings to maintain the quality of registering.

Studies involving FaMe-Net data are exempted from ethical review by the CCMO (Dutch Central Committee on research involving human subjects). Patients are extensively informed about including their health-related information in FaMe-Net, and are offered the opportunity to opt out of FaMe-Net [15].

### Participants and procedure

From 2016 onwards, patients from whom an email address is known received an online questionnaire that addressed contextual factors. Each patient received this questionnaire only once in the period 2016–2019, but not all simultaneously. One of the questions in the questionnaire addressed abuse: ‘Are (were) you a victim of sexual, physical or psychological abuse?’ with the answer options ‘yes’ or ‘no’. More than half of the enrolled patients completed this questionnaire.

We aimed to analyse all contacts with the GP in the past 5 years. To this end, we extracted data in the period 2015–2019 for all patients who filled out the questionnaire in this period, if they met the following conditions: aged ≥ 18 years in 2015 and registered in one of the participating practices for more than 1 year. The data extraction period, 2015–2019, was the same for all patients, independent of the date patients filled out the questionnaire.

### Variables

In this research, history of abuse includes both past and current abuse, abuse as a child as well as an adult and abuse by someone known or unknown. Furthermore, help-seeking behaviour is defined as how often a patient contacts the GP. Within FaMe-Net this translates into contact frequency. The reason why a patient contacts the GP is translated into RFEs, episode diagnoses and a number of contacts for these diagnoses.

We extracted and analysed data on contact frequency, types of contact, the first RFE of an episode of care, the episode diagnosis, and all contacts for an episode diagnosis. The types of contacts were clustered in (1) any type (phone, email, visit, consult) of contact with the GP and (2) any type of contact with the emergency GP during out-of-hours. The RFEs and diagnoses were clustered by the categories according to the ICPC, resulting in 17 categories (e.g. neurological, cardiovascular, psychological, etc.).

### Statistical analysis

Patient characteristics (gender, age in categories, country of origin (country of birth: Dutch or other)) were compared between the groups with and without a history of abuse, with two-by-two contingency tables. For checking of non-response bias, the same characteristics were compared between the non-response (who did not fill out the questionnaire) and response group, except for country of origin, which was unknown in the non-response group (country of origin was also a question in the questionnaire) (Supplementary Table 1).

A difference in the number of contacts, contacts with the GP and contacts with the emergency GP (out of office hours), RFEs and diagnoses between the abused and non-abused group were tested using a Negative binomial regression model. A negative binomial regression was performed because we expected over-dispersed count data for these outcomes. Gender, age categories, country of origin (Dutch or other) and practice were used as confounders. The difference between the abused and non-abused groups is expressed as the rate ratio (RR). It compares the incidence rate of an outcome variable over a certain period among those with a specific exposure (abuse) to those who were not exposed. The effect between men and women was investigated by including an interaction term between gender and a history of abuse in the model. A non-significant interaction term indicates that the same RR applies to both men and women. A *p* < .05 was considered statistically significant, based on two-sided testing. All analyses were performed with SPSS version 25.0.

The ICPC categories for RFEs and episode diagnoses were ordered by RR and the top five is shown in the results, the results of the remaining categories are shown in Supplementary Tables 2–4. For the contacts per episode diagnoses, we used the same order of ICPC categories as we used for the episode diagnoses.

## Results

### Patient characteristics

The response rate of the questionnaire was 59%. This resulted in including 11,140 patients (42,121 patient-years), of which 1271 indicated a history of abuse (Table 1). About 7% of men and 15% of women indicated a history of abuse.

**Table 1.** Characteristics of the included study group of abused and non-abused patients.

	Abused *n* = 1271 (11.4%)	Non-abused *n* = 9869 (88.6%)	All *n* = 11,140 (100%)
**Gender**			
Male	353 (27.8%)	4606 (46.7%)	4959 (44.5%)
Female	918 (72.2%)	5263 (53.3%)	6181 (55.5%)
**Age categories**			
18–35 years	438 (34.5%)	3918 (39.7%)	4356 (39.1%)
36–54 years	551 (43.4%)	3236 (32.8%)	3787 (34%)
≥55 years	282 (22.2%)	2715 (27.5%)	2997 (26.9%)
**Country of origin**			
Native Dutch	997 (78.4%)	8000 (81.1%)	8997 (80.8%)
Other	274 (21.6%)	1869 (18.9%)	2143 (19.2%)
**Patient years**	4820 (mean 3.8)	37,302 (mean 3.8)	42,122 (mean 3.8)

The non-response group consisted of more men than women (51.8% men and 48.2% women), in contrast to the response group that consisted of more women than men (44.5% men and 55.5% women). Distribution within the age categories was comparable.

### Contacts

Overall, men and women with a history of abuse contacted the GP (in daily practice and emergency practice) 1.5 times more often than men and women without a history of abuse (Table 2). Men with a history of abuse contacted the GP about 9 times a year and women with a history of abuse about 14 times a year. All RRs of the different contact types are equal in both men and women.

**Table 2.** Number of contacts per patient-year in abused and non-abused patients by gender and type of contact.

	Abused Mean number per patient-year	Non-abused Mean number per patient-year	RR^a^	95% CI	*p* Value^b^
All contacts (in daily practice and emergency practice)					
Men	9.23	6.14	1.50	1.42–1.60	<.001
Women	13.73	9.13	1.50	1.42–1.60	<.001
All^c^	12.48	7.73			
All contacts GP in daily practice					
Men	7.49	5.11	1.47	1.38–1.56	<.001
Women	11.27	7.69	1.47	1.38–1.56	<.001
All^c^	10.22	6.49			
All contacts emergency GP					
Men	1.35	0.81	1.67	1.57–1.78	<.001
Women	1.89	1.13	1.67	1.57–1.78	<.001
All^c^	1.74	0.98			

^a^Rate ratio.

^b^Wald Chi-square.

^c^Rate ratios for ‘All’ row not presented.

### Reason for encounter

Overall, men and women with a history of abuse had a significantly higher number of RFEs compared to men and women without a history of abuse, except for the ICPC categories ‘hearing system,’ ‘pregnancy and related problems,’ ‘male reproductive system’ and ‘blood and immune system problems.’

For both men and women with a history of abuse the RFE was almost twice more often for psychological problems (RR 1.97; *p* ≤ .001) and social problems (RR 1.93; *p* ≤ .001) compared to men and women without a history of abuse (Table 3). Digestive, endocrine and neurological symptoms were 1.5 times more frequent in men and women with a history of abuse.

**Table 3.** Number of reasons for encounters clustered by categories per patient-year for abused and non-abused patients by gender.

ICPC categories	Abused Mean number per patient-year	Non-abused Mean number per patient-year	RR^a^	95% CI	*p* Value^b^
Psychological problems					
Men	0.16	0.08	1.97	1.79–2.17	<.001
Women	0.21	0.11	1.97	1.79–2.17	<.001
All^c^	0.20	0.10			
Social problems					
Men	0.05	0.03	1.93	1.68–2.22	<.001
Women	0.09	0.04	1.93	1.68–2.22	<.001
All^c^	0.08	0.04			
Digestive organs					
Men	0.23	0.15	1.52	1.39–1.65	<.001
Women	0.36	0.24	1.52	1.39–1.65	<.001
All^c^	0.32	0.20			
Endocrine metabolic and nutritional problems					
Men	0.05	0.03	1.51	1.29–1.77	<.001
Women	0.07	0.04	1.51	1.29–1.77	<.001
All^c^	0.06	0.04			
Neurological system					
Men	0.10	0.07	1.43	1.28–1.59	<.001
Women	0.14	0.10	1.43	1.28–1.59	<.001
** **All^c^	0.13	0.09			

^a^Rate ratio.

^b^Wald Chi-square.

^c^Rate ratios for ‘All’ row not presented.

### Diagnoses

The highest differences in diagnoses between patients with and without a history of abuse are for psychological problems (RR 1.98) and social problems (RR 1.94), both almost twice as much in men and women with a history of abuse (Table 4). Neurological, digestive and endocrine problems are diagnosed about 1.5 times more often in men and women with a history of abuse.

**Table 4.** Number of episode diagnoses clustered by categories per patient-year for abused and non-abused patients by gender.

ICPC categories	Abused Mean number per patient-year	Non-abused Mean number per patient-year	RR^a^	95% CI	*p* Value^b^
Psychological problems					
Men	0.17	0.09	1.98	1.80–2.17	<.001
Women	0.22	0.11	1.98	1.80–2.17	<.001
All^c^	0.21	0.10			
Social problems					
Men	0.07	0.03	1.94	1.70–2.21	<.001
Women	0.11	0.05	1.94	1.70–2.21	<.001
All^c^	0.10	0.04			
Neurological system					
Men	0.09	0.09	1.51	1.34–1.69	<.001
Women	0.13	0.06	1.51	1.34–1.69	<.001
All^c^	0.12	0.07			
Digestive organs					
Men	0.23	0.15	1.50	1.38–1.63	<.001
Women	0.34	0.23	1.50	1.38–1.63	<.001
All^c^	0.31	0.19			
Endocrine, metabolic and nutritional problems					
Men	0.05	0.03	1.47	1.27–1.71	<.001
Women	0.07	0.05	1.47	1.27–1.71	<.001
All^c^	0.06	0.04			

^a^Rate ratio.

^b^Wald Chi-square.

^c^Rate ratios for ‘All’ row not presented.

Table 5 shows the number of contacts for each episode of care clustered by ICPC categories for patients with and without a history of abuse. For the ICPC categories, psychological, social, neurological, digestive organs and nutritional problems, the number of contacts was one to three times higher for men and women with a history of abuse than patients without a history of abuse. For the psychological and endocrine metabolic and nutritional problems the RR differed for men and women.

**Table 5.** Number of contacts per episode diagnoses clustered by categories per patient-year for abused and non-abused patients by gender.

ICPC categories	Abused Mean number per patient-year	Non-abused Mean number per patient-year	RR^a^	95% CI	*p* Value^b^
Psychological problems					
Men	2.03	0.60	3.38	3.00–3.81	<.001
Women	2.32	0.82	2.83	2.62–3.05	<.001
All^c^	2.24	0.72			
Social problems					
Men	0.21	0.10	2.14	1.97–2.34	<.001
Women	0.42	0.19	2.14	1.97–2.34	<.001
All^c^	0.36	0.15			
Neurological system					
Men	0.28	0.16	1.79	1.64–1.94	<.001
Women	0.44	0.25	1.79	1.64–1.94	<.001
All^c^	0.40	0.21			
Digestive organs					
Men	0.63	0.41	1.53	1.43–1.65	<.001
Women	0.93	0.61	1.53	1.43–1.65	<.001
All^c^	0.85	0.52			
Endocrine metabolic and nutritional problems					
Men	0.50	0.47	1.08	0.93–1.25	.338
Women	0.74	0.56	1.32	1.20–1.45	<.001
All^c^	0.67	0.52			

^a^Rate ratio.

^b^Wald Chi-square.

^c^Rate ratios for ‘All’ row not presented.

Men with a history of abuse contacted the GP 3.38 times more often per year for a psychological episode of care than men without a history of abuse; it was 2.83 times more often for women. In case of a social episode of care, both men and women with a history of abuse contacted the GP 2.14 times more frequently.

## Discussion

### Main findings

To our knowledge, this is the first study indicating that not only women with a history of abuse but also men with a history of abuse contact their GP 1.5 times more often than women and men without a history of abuse. For men and women with a history of abuse the reason for encounter was almost twice more often psychological problems and social problems than patients without a history of abuse, and the GP diagnosed a psychological or social problem almost two times more often. When diagnosed, men with a history of abuse more frequently contact the GP for psychological, social, neurological, digestive organs and nutritional problems than men without a history of abuse. This ratio is even larger for men that for women.

### Strengths and limitations

A significant strength of this study is that the data has been extracted from a long-lasting, reliable registration network, FaMe-Net. This is a network in which the participating GPs have regular meetings to maintain the quality of registering and have clear agreements for coding RFEs, diagnoses and interventions. Even though the data source is somewhat limited due to only five practices contributing data, it is reassuring that the coding quality is maintained. The age and gender distribution in the FaMe-Net database represents general practices across the Netherlands [17]. However, generalisability outside of the Netherlands is limited since cultural differences play a role in help-seeking behaviour of men and women. Another strength is a large number of included patients and contacts, allowing for corrections for confounders without losing statistical power. A large number of included patients and contacts ensures that minor differences in values lead to significant differences, which is statistically very strong but may lead to results less relevant in clinical settings. Nevertheless, we consider our findings relevant for daily care.

There are limitations and strengths to the questionnaire question: ‘Are (were) you a victim of sexual, physical or psychological abuse?’ with the answer options ‘yes’ or ‘no.’ A strength is that we look at the effect of all types of psychological, physical and sexual abuse, which on one hand gives a general picture of the effect of abuse on help-seeking behaviour. On the other hand, one might consider this a limitation as it does not differentiate between the different types of abuse. It is known from the literature that not experiencing violence as such is decisive for the effects in later life, but instead contextual factors such as the onset of the abuse, duration, relation with the perpetrator and if any support was present [18]. Unfortunately our study lacks information on this. Another possible limitation to the questionnaire question is the question wording and the binary response options. These may lead to respondents interpreting the question on victimisation in different ways. Moreover, self-reporting bias due to gender may also lead to differences in answering the question on victimisation [19]. However, since we analyse the differences in help-seeking behaviour between patients with or without a history of abuse, self-reporting bias due to gender does not affect our investigation.

Lastly, an unknown number of abused patients did not fill out the questionnaire. The incidence of abuse in this study is lower than surveys in the Dutch general population, which may indicate selection bias in our study [20–22].

### Comparison with existing literature

In our study, about 7% of men and 15% of women indicated a history of abuse. A recent study in the Netherlands revealed that 6% of men and 22% of women experienced a history of sexual abuse, showing a lower male to female ratio than our study [18]. Two Swedish cross-sectional studies among 6000 men and 6000 women showed a lifetime prevalence of a history of abuse among men and women of 16.7% and 18.2% for emotional abuse, 48.9% and 19.4% for physical abuse and 4.5% and 9.2% for sexual abuse, respectively [21,22]. Extensive questionnaires about abuse were used in the aforementioned studies. This was not the case in our study, which may have resulted in patients not considering some incidents as abuse, resulting in an underrepresentation of victims. For example, it is common for victims of psychological abuse not to recognise that what they are experiencing is abuse. Furthermore, disclosing by email is not a safe place and current victims of IPV may not feel safe to answer yes in case their email may be checked or monitored. It is known that men contact the GP less often than women, this is in line with our findings. Men contact the GP six to nine times a year and women nine to fourteen times [13,23–25]. Explanations for men’s low help-seeking behaviour are that in conformity with masculinity roles they less easily share personal information, experience a sense of immunity and immortality, have difficulty relinquishing control and believe that seeking help is unacceptable [23–25]. After sexual abuse men report barriers for seeking help such as social (traditional gender roles and norms), personal (shame, identity impacts) and practical barriers [13,26]. These explanations and barriers indicate that men find it more challenging to open up and discuss abuse, resulting in a lower contact frequency for men with a history of abuse than women with a history of abuse.

As research has shown that a history of abuse can lead to adverse long-term effects on mental and physical health for both men and women, the higher contact frequency found in our study was to be expected [1–9]. Victims of partner violence consult their GP more often, and visit the GP also more often for social problems [5,6]. Victims of childhood sexual abuse were more likely to experience psychiatric and behavioural problems, and have a higher contact frequency with public mental health services than non-childhood sexual abuse victims [27]. This is in line with findings in our study. Diagnoses for psychological and social problems are almost twice as often made in the group with a history of abuse, and when diagnosed with psychological and social problems, patients with a history of abuse, especially men, contact the GP more than twice as often for these problems.

## Conclusion

Our study shows that a history of abuse not only affects the help-seeking behaviour of women but also that of men. Men with a history of abuse, just like women with a history of abuse, contact the GP more often and suffer from more psychological and social problems. The increase in contacts, especially for psychological and social problems, should alert GPs to inquire about a history of abuse. Clinical enquiry alone does not necessarily improve the outcome of patients experiencing past or current abuse. We therefore strongly recommend appropriate training and specialist advocacy support provision. The management of affected patients requires a close relationship between GPs and practice teams on the one hand, and specialist agencies on the other, linking primary care into an intersectional response to violence and abuse [28].

## Supplementary Material

Supplemental Material
