# Supplementary material for: Help-seeking behaviour in primary care of men and women with a history of abuse: A Dutch cohort study
Source: Eur J Gen Pract. 2022 Apr 5;28(1):40–7. doi: 10.1080/13814788.2022.2054985 (PMC8986289; doi:10.1080/13814788.2022.2054985)
Supplement: Supplemental Material [file IGEN_A_2054985_SM8846.docx]

Supplementary data

| Table S1. Characteristics of the included study group of abused and non-abused patients, and of the excluded group. | | | | |
| --- | --- | --- | --- | --- |
|  | **Abused n=1271 (11.4%)** | **Non-abused**  **n=9869 (88.6%)** | **Excluded n=14,493 (56,5%)** | **Included**  **n=11,140 (43,5%)** |
| Gender   - Male - Female | 353 (27.8%)  918 (72.2%) | 4606 (46.7%)  5263 (53.3%) | 7503 (51.8%)  6990 (48.2%) | 4959 (44.5%)  6181 (55.5%) |
| Age categories   - 18-35 year - 36-54 year - ≥55 year | 438 (34.5%)  551 (43.4%)  282 (22.2%) | 3918 (39.7%)  3236 (32.8%)  2715 (27.5%) | 5149 (35.5%)  5383 (37.1%)  3961 (27.2%) | 4356 (39.1%)  3787 (34.0%)  2997 (26.9%) |
| Country of origin   - Native Dutch - Other | 997 (78.4%)  274 (21.6%) | 8000 (81.1%) 1869 (18.9%) |  |  |
| Practice   - 1 - 2 - 3 - 4 - 5 | 448 (35.2%)  203 (16.0%)  404 (31.8%)  74 (5.8%)  142 (11.2%) | 3497 (35.4%)  1637 (16.6%)  3153 (31.9%)  538 (5.5%)  1044 (10.6%) | 4012 (27.7%)  2760 (19.0%)  3661 (25.3%)  1445 (10.0%)  2615 (18.0%) | 3945 (35.4%)  1840 (16.5%)  3557 (31.9%)  612 (5.5%)  1186 (10.6%) |
| Patient years | 4,820 (mean 3.8) | 37,302 (mean 3.8) | 59,529 (mean 4.1) | 42,122 (mean 3.8) |

| Table S2. Number of reason for encounters clustered by categories per patient year for abused and non-abused patients by gender. | | | | | |
| --- | --- | --- | --- | --- | --- |
| ICPC categories | **Abused** Mean number per patient year | **Non-abused**  Mean number per patient year | **RR** | **95% CI** | **P value ^a^** |
| Psychological problems  Men  Women | - 0.16 - 0.21 | - 0.08 - 0.11 | 1.97 | 1.79-2.17 | < 0.001 |
| Social problems  Men  Women | - 0.05 - 0.09 | - 0.03 - 0.04 | 1.93 | 1.68-2.22 | < 0.001 |
| Digestive organs  Men  Women | - 0.23 - 0.36 | - 0.15 - 0.24 | 1.52 | 1.39-1.65 | < 0.001 |
| Endocrine metabolic and nutritional problems  Men  Women | - 0.05 - 0.07 | - 0.03 - 0.04 | 1.51 | 1.29-1.77 | < 0.001 |
| Neurological system  Men  Women | - 0.10 - 0.14 | - 0.07 - 0.10 | 1.43 | 1.28-1.59 | < 0.001 |
| General and non-specified  Men  Women | - 0.37 - 0.51 | - 0.26 - 0.36 | 1.41 | 1.30-1.52 | < 0.001 |
| Musculo-skeletal system  Men  Women | - 0.48 - 0.55 | - 0.35 - 0.41 | 1.36 | 1.26-1.47 | < 0.001 |
| Respirator system  Men  Women | - 0.30 - 0.38 | - 0.23 - 0.29 | 1.32 | 1.21-1.43 | < 0.001 |
| Cardiovascular system  Men  Women | - 0.09 - 0.10 | - 0.07 - 0.08 | 1.27 | 1.12-1.43 | < 0.001 |
| Female reproductive system  Women | - 0.27 | - 0.22 | 1.23 | 1.11-1.36 | < 0.001 |
| Visual system  Men  Women | - 0.10 - 0.13 | - 0.09 - 0.11 | 1.19 | 1.06-1.32 | 0.002 |
| Skin  Men  Women | - 0.43 - 0.53 | - 0.37 - 0.45 | 1.18 | 1.09-1.27 | < 0.001 |
| Urinary tract system  Men  Women | - 0.08 - 0.29 | - 0.07 - 0.26 | 1.14 | 1.04-1.25 | 0.005 |
| Hearing system  Men  Women | - 0.17 - 0.15 | - 0.15 - 0.14 | 1.10 | 1.00-1.22 | 0.053 |
| Blood, blood forming organs and immune system  Men  Women | - 0.01 - 0.02 | - 0.01 - 0.02 | 1.10 | 0.85-1.43 | 0.477 |
| Pregnancy and pregnancy related problems  Women | - 0.02 | - 0.02 | 1.05 | 0.91-1.21 | 0.494 |
| Male reproductive system  Men | - 0.06 | - 0.06 | 1.04 | 0.81-1.33 | 0.756 |
| ^a^ Wald chi-square | | | | | |

| Table S3. Number of episode diagnoses clustered by categories per patient year for abused and non-abused patients by gender. | | | | | |
| --- | --- | --- | --- | --- | --- |
| ICPC categories | **Abused** number per patient year (95% CI) | **Non-abused**  number per patient year (95% CI) | **RR** | **95% CI** | **P value ^a^** |
| Psychological problems  Men  Women | - 0.17 - 0.22 | - 0.09 - 0.11 | 1.98 | 1.80-2.17 | < 0.001 |
| Social problems  Men  Women | - 0.07 - 0.11 | - 0.03 - 0.05 | 1.94 | 1.70-2.21 | < 0.001 |
| Neurological system  Men  Women | - 0.09 - 0.13 | - 0.09 - 0.06 | 1.51 | 1.34-1.69 | < 0.001 |
| Digestive organs  Men  Women | - 0.23 - 0.34 | - 0.15 - 0.23 | 1.50 | 1.38-1.63 | < 0.001 |
| Endocrine metabolic and nutritional problems  Men  Women | - 0.05 - 0.07 | - 0.03 - 0.05 | 1.47 | 1.27-1.71 | < 0.001 |
| Female reproductive system  Women | - 0.23 | - 0.16 | 1.41 | 1.27-1.57 | < 0.001 |
| General and non-specified  Men  Women | - 0.34 - 0.56 | - 0.25 - 0.42 | 1.34 | 1.24-1.45 | < 0.001 |
| Musculo-skeletal system  Men  Women | - 0.46 - 0.53 | - 0.35 - 0.40 | 1.33 | 1.23-1.44 | < 0.001 |
| Respirator system  Men  Women | - 0.29 - 0.37 | - 0.22 - 0.29 | 1.30 | 1.20-1.41 | < 0.001 |
| Cardiovascular system  Men  Women | - 0.09 - 0.10 | - 0.07 - 0.08 | 1.26 | 1.12-1.42 | < 0.001 |
| Skin  Men  Women | - 0.46 - 0.55 | - 0.39 - 0.46 | 1.20 | 1.11-1.29 | < 0.001 |
| Visual system  Men  Women | - 0.10 - 0.12 | - 0.08 - 0.10 | 1.18 | 1.06-1.32 | 0.003 |
| Urinary tract system  Men  Women | - 0.07 - 0.30 | - 0.06 - 0.27 | 1.14 | 1.04-1.25 | 0.004 |
| Male reproductive system  Men | - 0.07 | - 0.07 | 1.09 | 0.86-1.38 | 0.480 |
| Hearing system  Men  Women | - 0.17 - 0.15 | - 0.16 - 0.14 | 1.08 | 0.98-1.19 | 0.135 |
| Pregnancy and pregnancy related problems  Women | - 0.03 | - 0.03 | 1.06 | 0.92-1.21 | 0.428 |
| Blood, blood forming organs and immune system  Men  Women | - 0.01 - 0.02 | - 0.01 - 0.02 | 0.99 | 0.78-1.25 | 0.923 |
| ^a^  Wald chi-square | | | | | |

| Table S4. Number of contacts per episode diagnoses clustered by categories per patient year for abused and non-abused patients by gender. | | | | | |
| --- | --- | --- | --- | --- | --- |
| ICPC categories | **Abused** number per patient year (95% CI) | **Non-abused**  number per patient year (95% CI) | **Rate ratio** | **95% CI** | **P value ^a^** |
| Psychological problems  Men  Women | - 2.03 - 2.32 | - 0.60 - 0.82 | 3.38  2.83 | 3.00-3.81  2.62-3.05 | < 0.001  < 0.001 |
| Social problems  Men  Women | - 0.21 - 0.42 | - 0.10 - 0.19 | 2.14 | 1.97-2.34 | < 0.001 |
| Neurological system  Men  Women | - 0.28 - 0.44 | - 0.16 - 0.25 | 1.79 | 1.64-1.94 | < 0.001 |
| Digestive organs  Men  Women | - 0.63 - 0.93 | - 0.41 - 0.61 | 1.53 | 1.43-1.65 | < 0.001 |
| Endocrine metabolic and nutritional problems  Men  Women | - 0.50 - 0.74 | - 0.47 - 0.56 | 1.08  1.32 | 0.93-1.25  1.20-1.45 | 0.338  < 0.001 |
| Female reproductive system  Women | - 0.62 | - 0.45 | 1.38 | 1.27-1.51 | < 0.001 |
| General and non-specified  Men  Women | - 0.83 - 1.31 | - 0.64 - 1.00 | 1.31 | 1.22-1.41 | < 0.001 |
| Musculo-skeletal system  Men  Women | - 0.95 - 1.38 | - 0.74 - 0.91 | 1.28  1.51 | 1.13-1.46  1.39-1.63 | < 0.001  < 0.001 |
| Respirator system  Men  Women | - 0.81 - 0.99 | - 0.57 - 0.70 | 1.41 | 1.32-1.51 | < 0.001 |
| Cardiovascular system  Men  Women | - 0.57 - 0.57 | - 0.50 - 0.49 | 1.15 | 1.07-1.25 | 0.001 |
| Skin  Men  Women | - 0.96 - 1.13 | - 0.79 - 0.93 | 1.22 | 1.14-1.30 | < 0.001 |
| Visual system  Men  Women | - 0.17 - 0.22 | - 0.14 - 0.18 | 1.21 | 1.10-1.33 | < 0.001 |
| Urinary tract system  Men  Women | - 0.20 - 0.60 | - 0.17 - 0.50 | 1.20 | 1.11-1.30 | < 0.001 |
| Male reproductive system  Men | - 0.22 | - 0.19 | 1.11 | 0.94-1.32 | 0.216 |
| Hearing system  Men  Women | - 0.29 - 0.26 | - 0.26 - 0.23 | 1.12 | 1.03-1.22 | 0.008 |
| Pregnancy and pregnancy related problems  Women | - 0.09 | - 0.08 | 1.15 | 1.04-1.28 | 0.008 |
| Blood, blood forming organs and immune system  Men  Women | - 0.02 - 0.17 | - 0.06 - 0.10 | 0.41  1.60 | 0.27-0.62  1.41-1.81 | < 0.001  < 0.001 |
| ^a^ Wald chi-square | | | | | |
